# Mode of action studies confirm on-target engagement of lysyl-tRNA synthetase inhibitor and lead to new selection marker for *Cryptosporidium*

**DOI:** 10.3389/fcimb.2023.1236814

**Published:** 2023-08-04

**Authors:** Jack C. Hanna, Victor Corpas-Lopez, Simona Seizova, Beatrice L. Colon, Ross Bacchetti, Grant M. J. Hall, Emma M. Sands, Lee Robinson, Beatriz Baragaña, Susan Wyllie, Mattie C. Pawlowic

**Affiliations:** ^1^Wellcome Centre for Anti-Infectives Research, School of Life Sciences, University of Dundee, Dundee, United Kingdom; ^2^Drug Discovery Unit, School of Life Sciences, University of Dundee, Dundee, United Kingdom

**Keywords:** cryptosporidiosis, mode of action, aminoacyl-tRNA synthtase, tRNA synthetase inhibitor, selection marker, genetic cross, thermal proteome profiling (TPP)

## Abstract

**Introduction:**

Cryptosporidiosis is a leading cause of diarrheal-associated morbidity and mortality, predominantly affecting children under 5 years old in low-and-middle-income countries. There is no effective treatment and no vaccine. New therapeutics are emerging from drug discovery efforts. It is critical that mode of action studies are performed alongside drug discovery to ensure the best clinical outcomes. Unfortunately, technology to identify and validate drug targets for *Cryptosporidium* is severely lacking.

**Methods:**

We used *C. parvum* lysyl-tRNA synthetase (*Cp*KRS) and DDD01510706 as a target-compound pair to develop both chemical and genetic tools for mode of action studies for *Cryptosporidium*. We adapted thermal proteome profiling (TPP) for *Cryptosporidium*, an unbiased approach for target identification.

**Results:**

Using TPP we identified the molecular target of DDD01510706 and confirm that it is *Cp*KRS. Genetic tools confirm that *Cp*KRS is expressed throughout the life cycle and that this target is essential for parasite survival. Parasites genetically modified to over-express *Cp*KRS or parasites with a mutation at the compound-binding site are resistant to treatment with DDD01510706. We leveraged these mutations to generate a second drug selection marker for genetic modification of *Cryptosporidium*, KRS^R^. This second selection marker is interchangeable with the original selection marker, Neo^R^, and expands the range of reverse genetic approaches available to study parasite biology. Due to the sexual nature of the *Cryptosporidium* life cycle, parental strains containing different drug selection markers can be crossed *in vivo*.

**Discussion:**

Selection with both drug markers produces highly efficient genetic crosses (>99% hybrid progeny), paving the way for forward genetics approaches in *Cryptosporidium*.

## Introduction

Diarrheal disease is responsible for 10% of the deaths of children under the age of five (Liu et al., 2012). The Global Enteric Multicenter Study (GEMS) determined that cryptosporidiosis is second only to Rotavirus in causing moderate to severe diarrheal disease (Kotloff et al., 2013). Cryptosporidiosis is caused by infection with the apicomplexan parasite *Cryptosporidium*, specifically *C. parvum* and *C. hominis* in humans, and over 30 other species in animals (Ryan et al., 2021). Cryptosporidiosis has profound impact in low-and-middle income countries, with the highest incidence observed in Sub-Saharan Africa (Khalil et al., 2018). In 2016, cryptosporidiosis caused an estimated 48,000 deaths of mostly young, malnourished children. Cryptosporidiosis is estimated to cause loss of 4.2 million disability adjust life years (DALYs) and an additional 7.85 million DALYs (Khalil et al., 2018) when compounded by malnutrition and growth stunting (Checkley et al., 1998; Moore et al., 2010). Immunocompromised adults, such as HIV/AIDS patients (Navin et al., 1999; O'Connor et al., 2011) and organ transplant recipients (Lanternier et al., 2017), are also at high risk of chronic, deadly cryptosporidiosis. There are no vaccines available to prevent cryptosporidiosis, and treatment options are extremely limited. Nitazoxanide, the only FDA approved treatment, is not effective in the patient populations most at risk of fatal *Cryptosporidium* infection—malnourished children and immunocompromised adults (Amadi et al., 2002; Amadi et al., 2009). In the absence of effective treatment options, there is great need to develop new anti-cryptosporidial drugs (Checkley et al., 2015).

GEMS revealed a greater global impact of cryptosporidiosis than was previously understood and inspired new anti-cryptosporidial drug discovery programs (Manjunatha et al., 2016). Many groups have repurposed advanced compounds active against *Plasmodium*, the causative agent of malaria, as a starting point for anti-cryptosporidial drug discovery (Love and Choy, 2021). One successful example of this strategy is provided by the repurposing of compounds designed to target *P. falciparum* lysyl-tRNA synthetase (KRS). The secondary fungal metabolite, cladosporin, was identified from a *Plasmodium* phenotypic screen and mode of action work determined its target to be *Pf*KRS (Hoepfner et al., 2012). Due to low metabolic stability of cladosporin, a biochemical screen against recombinant *Pf*KRS protein was performed to identify inhibitors with alternate chemotypes. A hit compound from this screen and analogues identified during the hit to lead optimization phase were found to be active against *Cryptosporidium* (Baragana et al., 2019). Treatment with “DDD01510706,” the early lead from this chemical series, in both the acute and the chronic cryptosporidiosis mouse models, reduces parasite shedding by two orders of magnitude. This established strong evidence of chemical validation of *Cp*KRS as a viable drug target for cryptosporidiosis.

Performing mode of action studies alongside drug discovery is essential for mitigating against drug resistance (Hasan et al., 2021) and populating the drug discovery pipeline with compounds of established, diverse modes of action (Huston et al., 2015; Katsuno et al., 2015). To date, there are very limited examples of mechanism of action studies for compounds in development for the treatment of cryptosporidiosis, including those targeting *Cp*KRS. For phenotypic hits, orthogonal screens aid in categorizing mode of action (Jumani et al., 2019), but do not identify the molecular target. Several compounds in development for cryptosporidiosis do not have a predicted or established target (Love et al., 2017; Janes et al., 2018; Stebbins et al., 2018).

Unbiased technologies for target identification in *Cryptosporidium* are critically important for advancing compounds closer to the clinic. *In vitro* evolution coupled with whole genome sequencing has proven incredibly powerful for identification of *P. falciparum* drug targets (Luth et al., 2018), but lack of a robust, simple, continuous *in vitro* culture for *Cryptosporidium* means this approach is still out of reach. Advances in chemical proteomic techniques offer an alternative in thermal proteome profiling (TPP), which has been used successfully to identify kinetoplastid and apicomplexan drug targets (Corpas-Lopez et al., 2019; Dziekan et al., 2019; Milne et al., 2022).

When a target is known, genetic validation is critical to fully understand the kinetics of cell death following target inhibition. This information is essential to prioritize/deprioritize drug discovery efforts (Forte et al., 2021). This is underscored in *Cryptosporidium* specifically by the genetic invalidation of both the classical apicomplexan target dihydrofolate reductase (DHFR) and also the promising target inosine monophosphate dehydrogenase (IMPDH) due to their metabolic redundancy in *Cryptosporidium* (Pawlowic et al., 2019). CRISPR/Cas9 can also be used to confirm that drugs are on-target. For example, edited versions of *C. parvum* phenylalanyl-tRNA synthetase (Vinayak et al., 2020) and methionyl-tRNA synthetase (Hasan et al., 2021) harboring mutations at the active site that impede compound binding are >20-fold or >100-fold resistant to their respective inhibitor, compared to wild type.

To address the need for mode of action tools to support drug discovery for cryptosporidiosis, we used *C. parvum* lysyl-tRNA synthetase and DDD01510706 as a target-compound pair to develop genetic and chemical-proteomics based mode of action tools for *Cryptosporidium*. This includes the first use of thermal proteome profiling in *Cryptosporidium* for target identification. Using this target-compound pair was critical to optimizing and establishing this complex methodology for *Cryptosporidium*. This was followed by genetic approach to confirm target essentiality, and on-target activity using over-expression and resistance-conferring mutations. This array of chemical and genetic tools is easily adapted to support other targets and compounds in development for cryptosporidiosis, and to aid further development of *Cp*KRS-targeting compounds.

In addition, we have leveraged this target-compound pair to generate a much needed second selectable drug marker for genetic modification of *Cryptosporidium*. Separately, over-expression or mutation of *CpKRS* conferred resistance to DDD01510706 *in vitro*. We combined these resistance-conferring modifications and found that over-expression of the mutated version of *CpKRS* confers resistance to DDD01510706 *in vivo* and is sufficient for use as a selection marker. This new selection marker, KRS^R^, can be used interchangeably with the original selection marker, neomycin resistance (Neo^R^).

Because *Cryptosporidium* engage in genetic crossing as a fundamental part of their life cycle *in vivo*, we used these two selection markers in combination to select for growth of hybrid parasites. Selection and growth of hybrids was remarkably efficient (>99%). Hybrid parasites carry drug markers inherited from both parental strains (Neo^R^ and KRS^R^) at the sporozoite level. Two selection markers enable forward genetic approaches to investigate important and unstudied aspects of *Cryptosporidium* biology including virulence, host-specificity, speciation, and mechanisms of parasite sexual recombination. Development of chemical-genetics mode of action tools and a second selection marker are powerful advancements for *Cryptosporidium* drug discovery and for parasite discovery biology.

## Materials and methods

### Materials

Oligonucleotides were purchased from Sigma Aldrich. Other materials purchased from Fisher Scientific, New England Biolabs, or as specified. Batches of >97% purity DDD01510706 (see **Supplemental Figure 1**) were prepared at the University of Dundee as previously described (Baragana et al., 2019). DDD01510706 is available upon request from Dr. Pawlowic (https://mrcppureagents.dundee.ac.uk/). Wild type *Cryptosporidium parvum* (Iowa II strain) oocysts were purchased from Bunchgrass Farms (Idaho, USA). Paromomycin sulfate and trimethoprim (TMP) (catalog #FT47738) were purchase from Carbosynth. Plasmids were constructed using HiFi cloning (NEB) and DNA sequence confirmed by Sanger sequencing (Genewiz). mScarlet-I was codon-optimized for *Cryptosporidium parvum*, produced as a gBlock by Integrated DNA Technologies.

### Thermal proteome profiling

Oocysts were treated with 2-4% sodium hypochlorite and washed in PBS as previously described (Pawlowic et al., 2017). For each biological replicate, 2x10^9^ wild type oocysts were prepared as described above, resuspended in 0.2 mM sodium taurocholate, and incubated at 37°C overnight. Excysted oocysts were centrifuged at 16,000 x*g* at 4°C for 10 min and resuspended in 2 mL ice-cold lysis buffer: 0.8% w/v n-octyl-β-d-glucoside, 50 mM K^+^ phosphate buffer pH 7.0, 1 mM EDTA, 250 mM sucrose, 1x EDTA free complete, EDTA-free Protease Inhibitor Cocktail (Roche), 1 mM DTT, and 100 μM Na-Tosyl-Lys-chloromethylketone (TLCK). Parasites underwent 10 freeze-thaw cycles (liquid nitrogen to room temperature) followed by two rounds of nitrogen cavitation on ice at a minimum of 2000 psi (4639 Cell Disruption vessel, Parr Instruments). Lysates were centrifuged at 100,000 x*g* for 20 min at 4°C; protein concentration was determined by Bradford assay (Bio-Rad).

**Figure 1:**
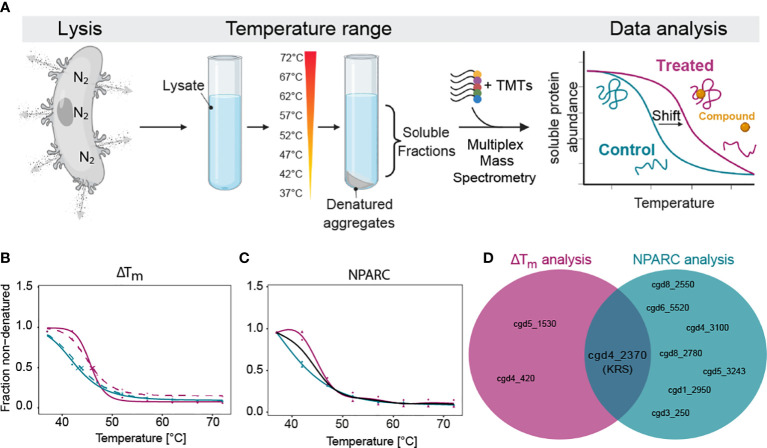
Thermal proteome profiling (TPP) determines that *Cp*KRS is the target of DDD01510706. **(A)** After excystation, *Cryptosporidium* sporozoites (wild type) were lysed using nitrogen cavitation. Lysate was incubated with or without DDD01510706 (10x EC_50_, 25 µM), aliquoted and subjected to a temperature range. Soluble proteins were precipitated and labelled with Tandem-Mass-Tags (TMTs), multiplexed, and analysed by mass spectrometry. Binding of a compound (yellow circle) to its protein target (correctly folded protein, magenta) commonly increases its thermal stability, often resulting in a measurable shift in the melting temperature (T_m_) of the target protein. **(B, C)** In the presence of DDD01510706, KRS (magenta) demonstrated a statistically relevant increase in thermal stability (+2.5°C) compared to control samples (teal) as determined by **(B)** ΔT_m_ analysis and **(C)** Non-Parametric Analysis of Response Curves (NPARC). **(D)** KRS is the only target identified by both analytic methods. Data represents 2 biological replicates with 1 technical replicate performed at each temperature. For ΔT_m_ analysis, solid and dashed line represent the two biological replicates. For NPARC analysis, solid line represents null model and colored lines represented experimental samples. Created with BioRender.com.

Lysate (minimum 0.45 mg/mL) was incubated with DDD01510706 at 10 x EC_50_ (25 µM), or equivalent volume DMSO (0.25% final concentration DMSO), for 30 min at room temperature. Samples were subdivided and incubated at a designated temperature (37-72°C) for 3 min, followed by room temperature for 3 min, and then incubated on ice. Samples were centrifuged at 100,000 x*g* for 20 min at 4°C. Soluble fractions were collected and protein concentration determined by Bradford assay. Two independent biological replicates with one technical replicate each were performed (**Figure 1A**).

Sample processing, TMT labelling (10-plex), fractionation by HPLC, LC-MS/MS analysis and protein identification and quantitation were performed as described previously except for the number of HPLC fractions, which was reduced from 24 to 10 fractions (Corpas-Lopez and Wyllie, 2021).

Proteins were identified using MaxQuant (http://maxquant.org/, version 2.0.1.0) (Cox et al., 2008) against the *C. parvum* Iowa II (Crypto DB version 46, cryptodb.org) (Amos et al., 2022). All MS data files have been deposited to the ProteomeXchange Consortium via the PRIDE partner repository with the identifier PXD037942 (Perez-Riverol et al., 2019).

### Molecular cloning and CRISPR design

Plasmids generated in this work are available upon request from Dr. Pawlowic (those of particular interest are available at https://mrcppureagents.dundee.ac.uk/). For imaging and conditional knockdown of *Cp*KRS (cgd4_2370), constructs with an mNeonGreen (*CpKRS*-mNG) or DDD tag (Choudhary et al., 2020) (*CpKRS*-DDD) were used for C-terminal fusion.

A red-shifted Firefly Luciferase-mNeon fusion was cloned into a plasmid containing a NanoLuciferase-Neomycin resistance fusion cassette (Nluc-Neo^R^). Both components are expressed under the control of *CpEnolase* regulatory elements to generate a “Δ*tk*::mNeon-Neo^R^” reporter strain (Firefly Luciferase, mNeonGreen, NanoLuciferase) (Vinayak et al., 2015; Costa et al., 2018). This Δ*tk*::mNeon-Neo^R^ strain serves as our NanoLuciferase-expressing WT *Cp*KRS strain used for *in vitro* drug assays, and also serves as our mNeon-Neo^R^ strain in the parasite cross.

*CpKRS* was cloned into a plasmid containing a NanoLuciferase-Neomycin resistance fusion cassette; both cassettes were expressed under *CpEnolase* regulatory elements to generate the “*Cp*KRS-OE” overexpression construct (Vinayak et al., 2015). To engineer the A309L amino acid substitution (*CpKRS-A309L*), the final 819 bp of *CpKRS* with the desired substitution was cloned as an mNeonGreen fusion reporter. To generate the novel drug marker cassette (KRS^R^), a NanoLuciferase-*CpKRS*-*A309L* fusion was cloned into the overexpression construct along with Firefly Luciferase and mScarlet-I (mScarlet-I codon optimized for *C. parvum*).

Guide RNAs (gRNA) were clones as previously described (Pawlowic et al., 2017). For all transgenics, 50 base pair regions of homology flanking the targeted loci were included on repair templates along with a Nluc-Neo^R^ or Nluc-KRS^R^ resistance marker.

To knockout *CpKRS* (Δ*krs*), a cassette with mNeonGreen was engineered to replace 86% of the gene. The 3’ region of *CpKRS* was targeted by a gRNA for knockout and tagging using the same guide RNA; for the A309L substitution, another guide targeting 256 base pairs upstream of the stop was employed. To generate the reporter Δ*tk*::mNeon-Neo^R^ strain or *CpKRS* over-expressor, the *CpTK* locus (cgd5_4440) was targeted by a gRNA sequence starting 15 base pairs upstream of the stop codon (Pawlowic et al., 2017). To generate the Δ*impdh*::mScarlet-KRS^R^ or Δ*impdh*::mNeon-Neo^R^ strains, a guide for the inosine monophosphate dehydrogenase (IMPDH cgd6_20) locus was used (Pawlowic et al., 2019).

### Propagation of transgenic *Cryptosporidium* in immunocompromised mouse models

All animal studies were ethically reviewed and carried out in accordance with Animals (Scientific Procedures) Act 1986 and the Dundee University Policy on the Care, Welfare, and Treatment of Animals. Regulated procedures on living animals were approved by the University’s Ethical Review Committee and carried out under the authority of project and personal licenses issued by the Home Office under the Animals (Scientific Procedures) Act 1986, as amended in 2012 (and in compliance with EU Directive EU/2010/63). The ERC has a general remit to develop and oversee policy on all aspects of the use of animals on University premises and is a subcommittee of the University Court, its highest governing body.

To generate new transgenic strains, Wild type *C. parvum* oocysts were excysted and transfected using a 4D Amaxa Nucleofector as previously described (Pawlowic et al., 2017). Female IFN-gamma KO mice (B6.129S7-Ifng^tmlTS^/J, JAX 002287; at least 8 weeks old) were given antibiotics in the drinking water for 1 week prior to infection. Mice were gavaged with saturated sodium bicarbonate 5 min prior to infection by gavage with transfected sporozoites. Paromomycin (16 g/L) and/or TMP (0.5 g/L) was added to the drinking water (made fresh three times a week) for the duration of the infection, or as indicated. Where indicated, mice were gavaged once daily for 7 days with 20 mg/kg DDD01510706 (prepared in 0.5% hydroxypropylmethylcellulose, 0.4% Tween 80 and 0.5% benzyl alcohol (v/v)).

IFN-gamma KO or NOD *scid* gamma mice (NOD.Cg-*Prkdc^scid^ Il2rgtm1Wjl*/SzJ; at least 6 weeks old) were used for propagating existing transgenic strains. Mice were gavaged with oocysts (single strain, or an equivalent number of Δ*impdh*::mScarlet-KRS^R^ and Δ*tk*::mNeon-Neo^R^ oocysts) and treated with selection agents as described above. Transgenic *Cryptosporidium* strains generated in this work are available upon request from Dr. Pawlowic and will be due to availability.

### Analysis of fecal samples

Fecal samples were collected at minimum every 2-3 days post infection, pooled from all mice in the cage, and parasite shedding was determined by NanoLuciferase assay (GloMax Navigator, Promega) and/or qPCR (QuantStudio 3 Real-Time PCR System, Thermo Fisher) (Sateriale et al., 2019). For NanoLuciferase assays, 20 mg of fecal material was homogenized in 1 ml of lysis buffer. Fecal RLUs throughout represent luminescence reading from 2 mg (0.1 ml) of fecal sample. DNA from infected fecal samples was extracted with the Quick-DNA Fecal/Soil Microbe Miniprep Kit (Zymo Research). Confirmation of successful modification of CRISPR-targeted loci was determined for each transgenic strain by PCR. Oocysts in fecal material were isolated using sucrose and cesium chloride flotations as previously described (Pawlowic et al., 2017).

### Microscopy

IFN-gamma KO mice were infected, and intestinal tissue harvested at peak infection (day 13 post infection). Intestines were flushed with PBS and fixed overnight in 4% paraformaldehyde at 4°C. Samples were dehydrated in 30% (w/v) sucrose, embedded in OCT compound (Agar Scientific) and cryosectioned (10 µm sections; Leica CM1850 cryostat). Cross-sections of intestine were stained with *Cp*TrpB (Striepen Lab), DAPI, and phalloidin-647 (Abcam, catalog #ab176759). Images were acquired using a Zeiss LSM 880 with Airyscan detector.

Live oocysts were imaged using a µ-Slide angiogenesis dish (Ibidi, catalog #81506) with a Leica Stellaris 8 Confocal microscope. Images of unexcysted oocysts were obtained by mounting bleached and washed oocysts in a 1:1 mix of Matrigel:PBS. Excysted oocysts were prepared by resuspending in a 1:1 mix of Matrigel:excystation buffer (0.25% trypsin, 0.05% taurodeoxycholic acid) (Zhang and Zhu, 2020) and imaged at 37°C.

All images collected at the Dundee Imaging Facility; The Open Microscope Environment (OMERO) was utilized for image management (https://www.openmicroscopy.org/omero/) (Allan et al., 2012). Videos and related images processed using Fiji 1.52i.

### *In vitro Cryptosporidium* culture

HCT-8 cells (ATCC catalogue #CCL-244; RRID: *CVCL_2478*) were cultured in 12-well (RT-qPCR) or 96-well plates (10,000/well for rate-of-kill assays; 20,000/well for drug assays) at 37°C, 5% CO_2_ as previously described (Pawlowic et al., 2017). HCT-8s were seeded 24 hours prior to infection; 5,000 - 20,000 transgenic oocysts were added per well. Compounds were prepared in 100% DMSO and were added at the time of infection (1% final concentration DMSO). Parasite growth was measured at the indicated time point (or at 48 hours post infection) by NanoLuciferase activity (GloMax Navigator, Promega) and host cell survival was measured using Cell Titer-Glo Luminescent Cell Viability Assay (Promega).

### *CpKRS* expression by RT-qPCR

Wild type or *Cp*KRS-OE oocysts were treated with 2-4% sodium hypochlorite and washed in PBS as previously described (Pawlowic et al., 2017). Oocysts were incubated with HCT-8s for two hours to allow infection, and then were washed vigorously with PBS to remove unexcysted oocysts. At various time points 3-72 hours post infection, total RNA was harvested and gDNA removed with the RNeasy mini kit (Qiagen) followed by cDNA synthesis (Lunascript RT SuperMix, NEB). Expression of *CpKRS* was determined relative to 18S rRNA by ΔΔC_T_ method. Expression at each timepoint was plotted relative to wild type expression at 3 hours post infection.

### Flow cytometry analysis

Fluorescent oocysts were bleached, washed with PBS, and filtered through a 40 μM filter. Laser intensities and gating strategy based on analysis of single strains for mNeon and mScarlet respectively, Δ*tk*::mNeon-Neo^R^ or Δ*impdh*::mScarlet-KRS^R^. A minimum of 50,000 events were recorded per sample. Samples were analyzed at the Flow Cytometry and Cell Sorting Facility in the School of Life Sciences, University of Dundee. FlowJo v10.7.1 was used for data analysis and visualization.

### Statistics

EC_50_ values and growth curve regressions (one-phase decay, non-linear regression for rate-of-kill assay) were calculated in GraphPad Prism v 9.2.0 from the dose response curves. Curves were tested using extra-sum-of-squares F test, where the null hypothesis (one curve fits both datasets) is rejected if p<0.05.

TPP sample analysis was performed using the Bioconductor TPP package as previously described (Franken et al., 2015; Corpas-Lopez et al., 2019; Corpas-Lopez and Wyllie, 2021). Briefly, reporter ion intensities were transformed into relative protein abundance by normalizing them to the lowest temperature. Melting curves were fitted and the melting point was determined using the TPP package. Then the melting point differences (ΔT_m_) were calculated and their statistical significance were calculated using a z-test where only proteins with an FDR-adjusted *p*-value <0.1 and <0.2 in the two biological replicates, respectively were considered hits. In addition, non-parametric analysis of response curves (NPARC) was performed (Childs et al., 2019). NPARC evaluates whether the experimental data fits better to a null model (that assumes that the treatment has no effect on the protein stability) or to an alternative model (that assumes that the treatment affects the protein stability). Proteins that significantly fit better to the alternative model can be selected as hits based on the FDR-adjusted *p*-value generated in the test.

## Results

### TPP identifies *Cp*KRS as the molecular target of DDD01510706

Originally developed as an anti-malarial, DDD01510706 is an early lead compound that is also active against *Cryptosporidium* (**Supplemental Figure 1**) (Baragana et al., 2019). DDD01510706 is effective in both acute and chronic cryptosporidiosis mouse models. When mice were treated at doses higher than those required for efficacy (20 mg/kg daily for 7 days), toxicity was observed. Although DDD01510706 is not being progressed due to this issue, it is an ideal drug-like tool compound for mode of action studies. Recent mode of action work confirmed that the target of DDD01510706 in *Plasmodium* is *PfKRS* (Milne et al., 2022), however, mode of action work is yet to be conducted in *Cryptosporidium*.

Based on the high degree of homology between *PfKRS* and *CpKRS* (47.7% sequence identity and 64.6% similarity), it is presumed that DDD01510706 will target KRS in *Cryptosporidium*. We adapted TPP for use in *Cryptosporidium*, an unbiased approach, to identify the molecular target of this compound (**Figure 1A**). TPP takes advantage of the fact that, in general, binding of a drug to its protein target can significantly alter the thermal stability of that protein (Savitski et al., 2014). To monitor this phenomenon, aliquots of compound-treated and control cell lysate are exposed to a range of temperatures. Soluble protein from each sample aliquot is harvested, labelled with Tandem-Mass-Tags (TMTs) to enable quantitation, and melting curves for each protein are established by mass spectrometry. Proteins that exhibit a significant and reproducible shift in thermal stability in the presence of the inhibitor are short listed as potential molecular targets. Thus, TPP can be used as an effective and unbiased approach to demonstrate compound-target engagement and has been used successfully in studies with a number of protozoan parasites including *Plasmodium* (Dziekan et al., 2019), *Leishmania* (Corpas-Lopez et al., 2019), and *Toxoplasma* (Herneisen et al., 2020).

Unlike other eukaryotic pathogens which can be cultured in large volumes, for *Cryptosporidium* we require large animal infections to generate sufficient parasite material for proteomics analysis. After much optimiziation, we were able to generate parasite lysate (wild type sporozoites) of sufficient quantity and quality. First, we excyst oocysts, then expose them to ten rounds of freeze-thaw to open any remaining unexcysted oocysts, and then lyse the free sporozoites by nitrogen cavitation. Nitrogen gas dissolves into solution under high pressure and diffuses across the parasite membranes. When the pressure is released, the nitrogen gas escapes, in the process lysing cells. Sporozoite lysate was divided into two and incubated with either DDD01510706 (at 10x EC_50_, 25 µM) or DMSO (control, 0.25% final concentration). Treated and control samples were aliquoted and exposed to one of a range of temperatures (37°C to 72°C). Soluble proteins from each temperature-exposed aliquot were harvested by centrifugation, prepared for mass spectrometry, multiplexed (10-plex) and analyzed.

As standard, our TPP datasets are analysed using two different statistical methods. The melting temperature (T_m_)-based method (Franken et al., 2015; Corpas-Lopez et al., 2019; Corpas-Lopez and Wyllie, 2021), assesses individual protein melt curves using several criteria including curve R squared, variability in T_m_ values within the control sample, maximum curve plateau and minimum slope. Melting point differences (T_m_, treated - T_m_, control = ΔT_m_) are then established for every detectable protein. The most significantly affected proteins are selected as potential “hits” by applying an FDR-adjusted z-test to ΔT_m_ data. Proteins with a *p*-value <0.1 in both technical replicates are considered “hits”. Unlike the T_m_ method of hit identification described above, NPARC considers the whole melting curve, comparing the goodness of fit of the experimental data to a null model that assumes that the protein is unaffected by drug treatment; or an alternative model that assumes that the protein is affected by the treatment (Childs et al., 2019). An FDR-adjusted *p*-value is generated that denotes the significance of the effect of the drug on protein melting behaviour. Proteins with an NPARC *p*-value <0.01 were considered “hits” and those hits common to both biological replicas were considered putative targets.

Melting curves were generated for 1857 proteins, representing 47.1% of the annotated *Cryptosporidium* genome (Baptista et al., 2022). In the presence of DDD01510706, three proteins were identified as potential targets by ΔT_m_ analysis (**Supplemental Table 1**; **Figure 1B**) while seven proteins were identified by NPARC (**Figure 1C**; **Supplemental Table 1**). However, *Cp*KRS was the only protein identified as a target by both statistical methods (**Figure 1D**). The identification of *Cp*KRS via this unbiased, proteome-wide approach, provides compelling evidence of a direct binding interaction between DDD01510706 and this enzyme. Thus, we have high degree of confidence that *Cp*KRS is the molecular target of this compound. To our knowledge, this represents the first use of TPP in *Cryptosporidium* and validates this approach for target identification of other anti-cryptosporidial compounds.

### Lysyl-tRNA synthetase (KRS) is an essential drug target in *Cryptosporidium*

**Figure 2:**
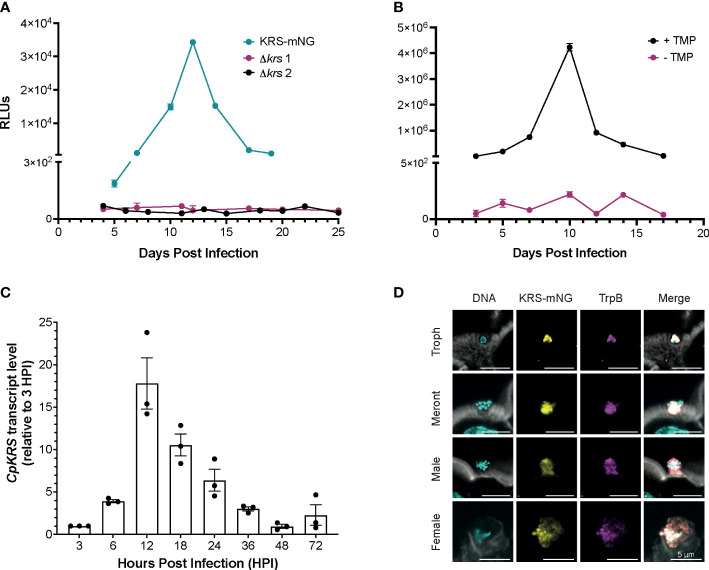
*Cryptosporidium* lysyl-tRNA synthetase (*CpKRS*) is an essential drug target. **(A)** *Cryptosporidium* sporozoites (wild type) were transfected with CRISPR/Cas9 constructs designed to target the 3´ region of the *KRS* gene for tagging with mNeonGreen (KRS-mNG, teal) or for gene knockout (Δ*krs*; two independent experiments, magenta and black). IFN-γ KO mice (4 per cage) were infected by gavage; fecal samples were collected from each cage and analyzed for expression of NanoLuciferase (RLUs, relative luminescence units; RLUs/2 mg of fecal material plotted; mean ± SD of three technical replicates). RLU >1000 indicate successful infection of transgenic parasites. **(B)** Mice were infected with a conditional *Cp*KRS *Cryptosporidium* strain (KRS-DDD). KRS is stabilized in the presence of TMP (trimethoprim delivered in the drinking water, black), and degraded in the absence of TMP (water only, magenta). Degradation of KRS in the absence of TMP prevents infection, as measured by RLU. Representative graph of 2 biological replicates performed, each with 3 technical replicates; mean ± SD at each time point. **(C)** Transcript levels of *CpKRS* quantified by RT-qPCR during *in vitro* infection (transcript abundance normalized to 18S rRNA levels at each timepoint and reported relative to *KRS* levels at 3 HPI). Mean ± SD with individual technical repeats indicated. Representative graph of 3 biological replicates performed, each with 3 technical replicates. **(D)** Immunofluorescence microscopy of intestines from mice infected with KRS-mNG from A (tissue collected day 13 post infection when infection peaked; actin stained with phalloidin, white; DNA stained with DAPI, cyan; KRS-mNG, yellow; cytoplasmic localized protein *Cryptosporidium* tryptophan synthase B, TrpB; magenta). KRS is expressed in asexual (single nuclei trophozoite, Troph; 8-nuclei Meront) and sexual stages. Scale bar 5 µm.

Following the identification of *Cp*KRS as the molecular target of DDD01510706, we aimed to complement previous chemical validation (Baragana et al., 2019) with a deeper understanding of the biology of this promising target. To genetically validate *CpKRS* as a drug target, we attempted to knockout the gene using CRISPR/Cas9 (**Supplemental Figure 2A**). Wild type sporozoites were transfected with guide RNA (gRNA) constructs designed to replace *CpKRS* with a repair cassette containing a NanoLuciferase-Neomycin resistance fusion (Nluc-Neo^R^) (Vinayak et al., 2015). Nluc-Neo^R^ allows for selection of transgenic parasites and monitoring of infection by NanoLuciferase activity in the fecal material. IFN-γ KO mice were infected, treated with the selection agent (paromomycin in the water bottle), and fecal samples were collected. In our model, relative luminescence units (RLU) above 1,000 indicates successful generation of transgenic parasites. In two independent experiments, we were unable to generate Δ*krs* parasites (**Figure 2A**). However, using the same wild type parasites and gRNA, a KRS-mNeonGreen strain was recovered (KRS-mNG; **Figure 2A**, **Supplemental Figures 2B, C**). Therefore, the genetic locus is amenable to modification. Inability to recover Δ*krs* parasites suggest that *CpKRS* is essential for parasite survival.

To further confirm essentiality, a conditional knockdown strain was designed. *Cp*KRS was fused at the C-terminus to a dihydrofolate destabilization domain (DDD; **Supplemental Figures 2D, E**). This system works such that the DDD fusion protein is stabilized and functional in the presence of trimethoprim (TMP), but in the absence of TMP the dihydrofolate domain is unfolded, destabilizing the fusion protein and targeting it for degradation by the parasite proteasome (Choudhary et al., 2020). We generated and maintained a KRS-DDD strain *in vivo* in the presence of TMP (supplied in the water bottle). It is difficult to measure *Cp*KRS-DDD knockdown by Western Blot, because it is difficult to prepare material from intracellular parasites stages, partially because *Cryptosporidium* parasites cannot be separated from their host cell. It is also difficult to validate knockdown by microscopy because epitope tagging of *Cp*KRS does not produce a bright enough signal to enable analysis. Therefore, we evaluated KRS knockdown *in vivo*, as previously described for another *Cryptosporidium* drug target (Choudhary et al., 2020). IFN-γ KO mice were infected with the KRS-DDD strain and infection patterns were observed in the presence or absence of TMP. When mice are treated with the stabilizing TMP, the infection follows the typical acute pattern (**Figure 2B**, black); in the absence of TMP no infection is observed (**Figure 2B**, magenta). Collectively, these data provide strong evidence that *CpKRS* is essential for *Cryptosporidium* infection.

Using RT-qPCR, we determined that *CpKRS* is transcribed, and presumably expressed, throughout the life cycle. Transcript levels are highest during the first 36 hours, during the asexual part of the life cycle (**Figure 2C**). Transcript levels peak at 12 hours, corresponding to the first round of merogony prior to egress (English et al., 2022). To determine subcellular localization, the KRS-mNG reporter strain was employed (**Figure 2A**). Tissue was collected from distal ileum of mice infected with KRS-mNG and analyzed by microscopy. We observed expression of *CpKRS* in all life cycle stages and a pattern consistent with the parasite cytoplasm, as indicated by co-localization with the cytoplasmic protein *Cryptosporidium* Tryptophan Synthase B (TrpB, **Figure 2D**).

### Over-expression or mutation of *CpKRS* confers resistance to DDD01510706

Another commonly employed approach to identify drug targets is to generate resistance and then deconvolve the genes involved in resistance. In *Plasmodium* this is done through *in vitro* evolution, where parasites are cultured in the presence of an inhibitor, eventually evolving resistance (Cowell et al., 2018). Resistant clones are subjected to whole genome sequencing, and single nucleotide polymorphisms (SNPs) or copy-number-variants often reveal the drug target. CRISPR/Cas9 is then used to engineer these resistance-conferring mutations to validate that they cause drug resistance. Although *in vitro* evolution is not yet possible for *Cryptosporidium*, if a target is known, CRISPR/Cas9 can be used to interrogate specific interactions with the compound of interest. Molecular targets can be overexpressed and/or mutated at key amino acid residues in the predicted binding site and the impact on compound susceptibility measured. With this in mind, we generated *Cryptosporidium* strains where *CpKRS* is modified and measured changes in susceptibility to DDD01510706. A strain over-expressing *Cp*KRS (KRS-OE) was generated by introducing an additional copy of *CpKRS* into the genome at an ectopic location (**Supplemental Figures 3A, D, G**). *CpKRS* expression, as determined by transcript abundance, was consistently higher in the KRS-OE strain relative to the parental wild type (**Supplemental Figure 4A**). Relative levels of *CpKRS* transcript were ≥ 3-fold higher than wild type at every time point, with the highest transcript levels at 12 hours, when expression of *Cp*KRS peaks.

**Figure 3:**
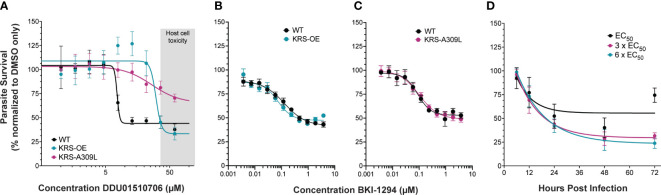
Genetic validation that KRS inhibitors are on-target. **(A)** Overexpression of *CpKRS* (KRS-OE, teal) or mutation of *CpKRS* with an A309L substitution (KRS-A309L, magenta) render parasites less susceptible to treatment with DDD01510706 compared to wild type parasites (WT, black). WT parasites used for drug assays are a transgenic reporter strain that expresses NanoLuciferase and are WT for *Cp*KRS (**Supplemental Figure 2C**). Host toxicity indicated in grey (see **Supplemental Figure 5**). **(B, C)** Drug mode of action is specific to inhibition of *Cp*KRS. KRS-OE and KRS-A309L are equally susceptible as WT parasites to treatment with compounds that inhibit a non-*Cp*KRS target (BKI-1294 an inhibitor of *Cp*CDPK1). Mean ± SEM; 4 biological replicates, each with 4 technical replicates. **(D)** WT parasites were cultured *in vitro* with DDD01510706 at the EC_50_ (7.5 µM, black), 3 x EC_50_ (22.5 µM, magenta), or 6 x EC_50_ (45 µM, teal) to determine the rate-of-kill. Compound was added at the time of infection, and parasite survival was measured by NanoLuciferase at the indicated timepoints post infection. Treatment with DDD01510706 reduces parasite growth within 24 hours post-infection, corresponding to peak *Cp*KRS expression. One phase decay linear regression plotted (GraphPad). Mean ± SD; representative graph of three biological replicates, each with 3 technical replicates. Curves were analyzed by extra-sum-of-squares F test, where the null hypothesis (one curve fits both datasets) is rejected if p<0.05. **Figure 1A**: WT vs OE-KRS p<0.0001; WT vs A309L-KRS p<0.0001. **Figures 1B, C**: WT vs OE-KRS p=0.1148; WT vs A309L-KRS p=0.8501

Studies in *Plasmodium* identified a DDD01510706 resistance-conferring mutation in the compound binding site of *PfKRS* (serine at position 344 to leucine) (Milne et al., 2022). We engineered the corresponding amino acid substitution (alanine 309 to leucine) at the endogenous *CpKRS* locus, to generate KRS-A309L (**Supplemental Figures 3B, E, H**; **Supplemental Figure 4B**). Wild type, KRS-OE, or KRS-A309L parasites were co-cultured with a human intestinal cell line *in vitro*. DDD01510706 was added at the time of infection and NanoLuciferase activity was measured 48 hours post infection to determine parasite survival. These drug sensitivity assays confirmed that both KRS-OE and KRS-A309L strains were resistant to DDD01510706 (**Figure 3A**) compared to the parental wild type. Specifically, KRS-OE was four-fold resistant to DDD01510706 (wild type EC_50_ = 7.5 µM; KRS-OE EC_50_ = 30.5 µM), similar to the levels of resistance demonstrated by the corresponding KRS overexpressing cell line in *Plasmodium* (Milne et al., 2022).

Unfortunately, DDD01510706 host toxicity (**Supplemental Figure 5**) prevented an accurate estimation of the level resistance conferred by the A309L mutation, however, it appears at least ≥ eight-fold resistant. Importantly, this resistance phenotype was specific for KRS inhibitors, since these strains did not demonstrate cross-resistance to an unrelated inhibitor of *Cp*CDPK1 (BKI-1294; **Figures 3B, C**) (Hulverson et al., 2017). Our *CpKRS* overexpression and mutation strains provide yet further evidence that KRS is the molecular target of DDD01510706 and suggest that the binding orientation of this compound is likely to mimic that seen in *Pf*KRS. In addition, these cell-based tools can be utilized to guide optimization of other compounds or series being developed for this promising molecular target.

*In vitro* rate of kill assays were performed to investigate the kinetics of inhibition (Jumani et al., 2019). Wild type *Cryptosporidium* parasites were cultured *in vitro* and DDD01510706 was added at the time of infection at 1x, 3x, or 6x EC_50_ (determined in **Figure 3A**). The impact of DDD01510706 on parasite growth was measured by NanoLuciferase assay at specific times post-infection, allowing the speed of compound-induced parasite death to be determined. DDD01510706 is fast-acting and exposure for as little as 12 hours results in a marked reduction in parasite growth, which increases to >50% kill by 24 hours (**Figure 3D**). This time frame corresponds to peak *CpKRS* expression. The kinetics of *Cp*KRS inhibition suggest parasiticidal activity, in stark contrast to the static effect of nitazoxaninde (Lunde et al., 2019).

### New selection marker, KRS^R^, expands the scope of *Cryptosporidium* reverse genetics and enables forward genetics

Using orthogonal strategies (TPP and genetic modification), we have validated that lysyl-tRNA synthetase is the specific molecular target of DDD01510706. Next, we replicated treatment of mice with DDD01510706 (20 mg/kg for 7 days, delivered by oral gavage) in the acute cryptosporidiosis model several times (**Supplemental Figure 6**). In each instance, we observed reduction of *Cryptosporidium* infection to near the limit of quantification, as previously reported. No toxicity was observed in animals dosed with DDD01510706 at this level since dosing was well within the selectivity window. Host toxicity at higher doses (≥ 20 mg/kg), has precluded the pre-clinical development of this compound for cryptosporidiosis and malaria. However, our increased knowledge of this target-compound pair, resulting from these mode of action studies, create a unique opportunity to leverage DDD01510706 as a tool compound.

**Figure 4:**
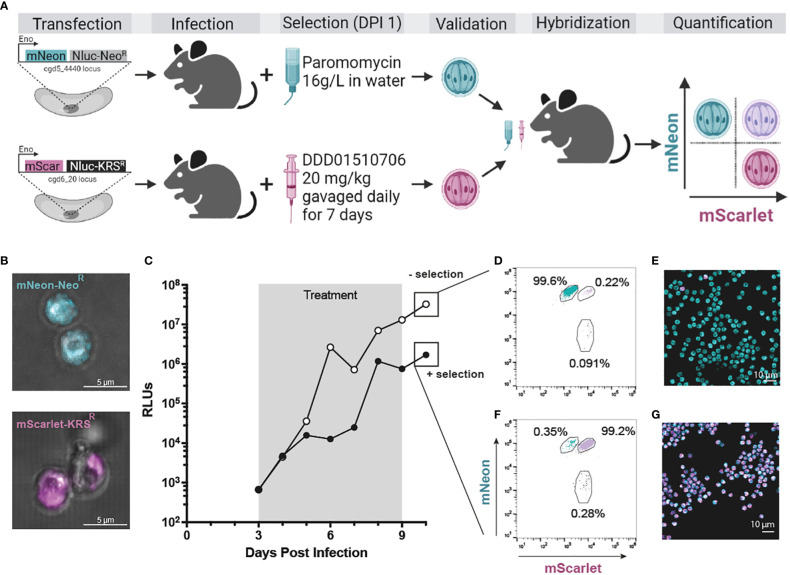
KRS^R^ is a new selection marker for *Cryptosporidium* genetic modification and hybridization. **(A)** *CpTK* locus was targeted for replacement with mNeon-Neo^R^ (teal; see **Supplemental Figure 3C** for CRISPR strategy). In a second strain *CpIMPDH* was targeted for replacement with mScarlet-KRS^R^ (magenta; see **Supplemental Figure 7B** for CRISPR strategy). Wild type sporozoites were transfected with either cassette and used to infect IFN-γ KO mice. Selection of transgenics began day 1 post infection (paromomycin in the water bottle or DDD01510706 by gavage, respectively; see **Supplemental Figure 8** for *in vivo* infection data). Stable transgenic strains were obtained (**Supplemental Figure 7D**; **Supplemental Figure 3F**) and utilized in a genetic crossing experiment (microscopy of oocysts of both strains **(B)**. Only hybrid parasites survive selection with both selection agents. Oocysts resulting from a genetic cross were quantified by flow cytometry and microscopy. An equal number of purified mNeon-Neo^R^ and mScarlet-KRS^R^ oocysts were used to infect two cages of mice. **(C)** One cage was treated with both selection agents (“+ selection”, grey box); control cage was treated with vehicle (“- selection”). Fecal samples were collected daily and infection measured by NanoLuciferase (RLUs, relative luminescence units; RLUs/2 mg of fecal material plotted). Oocysts were purified from fecal samples collected day 10 post infection. **(D, E)** Flow cytometry (50,000 events recorded). Flow cytometry was used to quantify the percentage of fluorescent oocysts from both selected and non-selected parasite population that express mNeon, mScarlet, or both (purple). **(E, G)** Purified oocysts were also analyzed by microscopy. Created with BioRender.com.

Overexpression of *CpKRS* and mutation of the *Cp*KRS active site confer resistance to DDD01510706 *in vitro* (**Figure 3A**). We evaluated if we could take advantage of these modifications of *CpKRS* to create a transgenic where the mutated version of *Cp*KRS (A309L) is overexpressed from a secondary locus, functioning as a selectable marker (**Figure 4A**). To do this, a construct was designed where the original selection marker (Neo^R^) was replaced with *CpKRS-A309L* (KRS^R^). In this new transgenic strain, the *Cp*KRS locus is not modified; KRS^R^ is overexpressed from a secondary locus—*CpIMDPH*. This KRS^R^ repair cassette also contains NanoLuciferase and mScarlet to quantify *in vivo* infection.

Wild type sporozoites were transfected with gRNA constructs designed to replace *CpIMPDH* with a repair cassette containing either an mScarlet-KRS^R^ or an mNeon-Neo^R^ repair cassette (**Supplemental Figures 7A, B**). IFN-γ KO mice were infected and treated with selection agent(s) starting at day 1 post infection for seven days total. This was administered as either paromomycin in the water bottle ("+ Paromomycin") or via oral gavage of DDD01510706 at 20 mg/kg once daily ("+ DDD01510706"). Fecal samples were collected and analyzed for presence of transgenic *Cryptosporidium* by NanoLuciferase assay and for overall quantification of *Cryptosporidium* infection by qPCR (**Supplemental Figure 8**). Each cage of mice became initially highly infected during the first four days post infection (as detected by qPCR) before the full effect of selection was observed (**Supplemental Figures 8E-H**).

We first confirmed the ability to modify the *CpIMPDH* locus (**Supplemental Figure 7C**). This locus was targeted for replacement with mNeon-Neo^R^ and paromomycin was used to select for the growth of transgenic parasites. Mice shed NanoLuciferase expressing parasites, confirming the ability to modify the *CpIMDPH* locus (**Supplemental Figures 8A**). Generation of mScarlet-KRS^R^ transgenic parasites was only possible when mice were treated with DDD01510706 as the selection agent (**Supplemental Figures 8D, H**; **Supplemental Figure 7D**). Parasites transfected with mScarlet-KRS^R^ but that were not treated with a selection agent did not become infected with transgenic parasites (RLU < 1000; **Supplemental Figures 8B, F**). Parasites transfected with mScarlet-KRS^R^ and that were treated with the wrong selection agent, paromomycin, also did not become infected with transgenic parasites (RLUs < 1000; **Supplemental Figures 8C, F**). The mScarlet-KRS^R^ strain was further passaged in mice (**Supplemental Figure 9**) and oocysts were purified from fecal material. Live microscopy of mScarlet-KRS^R^ oocysts confirmed mScarlet expression (**Figure 4B**). These studies validate KRS^R^ as the second selectable marker capable of supporting the genetic manipulation of *Cryptosporidium* (**Figure 4A**). Access to a second selection marker expands reverse genetic tools and has great potential to increase our understanding of the biology of this important parasite.

Unlike *Plasmodium*, after three rounds of asexual development, *Cryptosporidium* parasites commit to male or female fates (Tandel et al., 2019; English et al., 2022). When a host is infected with a single strain of *Cryptosporidium*, parasite gametes self-fertilise and complete the life cycle. However, when more than one strain of *Cryptosporidium* is present in the host, parasites can cross-fertilise and engage in sexual recombination (Feng et al., 2002). With only a single selection marker, we cannot select for growth of hybrids. Instead Mendelian inheritance is observed where only ~25% of progeny are hybrids (Wilke et al., 2019).

To test if two drug markers can be utilized to select for exclusive growth of hybrid parasites, we infected mice with equal numbers of mScarlet-KRS^R^ and mNeon-Neo^R^ transgenic oocysts and treated mice with both selection agents (**Figure 4A**). Parental strains used for this experiment were freshly passaged (<1 month prior to this infection). For this crossing experiment, mNeon-Neo^R^ strain is modified at the *CpTK* locus (**Supplemental Figure 2C**), and the mScarlet-KRS^R^ modification is introduced at the *CpIMPDH* locus on a different chromosome. Starting at day 3 post infection, after completion of one round of sexual reproduction, one cage received both selection agents for 7 days (**Figure 4C**, “+ selection” in black) and the second cage received no selection agents (**Figure 4C**, “- selection” in white). Oocysts were purified from fecal material collected day 10 post infection, after selection ended. The resulting oocyst population from each group was quantified by flow cytometry (gating strategy described in **Supplemental Figure 10**) and analyzed by microscopy (**Figures 4E, G**).

Mice that did not receive any selection agents shed < 0.5% hybrid parasites, and >99% of the parental mNeon^+^ strain (**Figures 4D, E**). This is due to differences in passage number and associated virulence of the parental strains (mNeon-Neo^R^ has been passaged in mice over 15 times and outcompetes mScarlet-KRS^R^, which has been passaged only 3 times). In contrast, mice treated with both selection agents shed > 99% hybrid parasites (mScarlet^+^ and mNeon^+^) and < 0.5% parental strain parasites (**Figure 4F**). This was confirmed by live microscopy of oocysts (**Figure 4G**). Hybrid oocysts were excysted and microscopy confirmed that sporozoites are themselves hybrid (**Supplemental Figure 11**). Used in combination, KRS^R^ and Neo^R^ produce highly efficient (>99%) *Cryptosporidium* experimental genetic crosses.

Further, KRS^R^ is interchangeable with Neo^R^ in DNA constructs and similarly can be used to modify any loci in the genome (mutation of the endogenous *CpKRS* gene is not required). In the case of crossing, simultaneous treatment with both selection agents is straightforward, and selection is strong enough to generate hybrids at the individual sporozoite level. Because progression through the sexual stage is required for completion of the life cycle, *Cryptosporidium* is extremely well-suited for studies involving genetic crosses. Forward genetics has proven a powerful tool to understand parasite biology for related apicomplexan parasites but requires two markers to select for hybrids. The creation of a second selection marker, KRS^R^, and the high efficiency of obtaining hybrid parasites forms the basis for forward genetic approaches in *Cryptosporidium*.

## Discussion

As cryptosporidiosis drug discovery efforts advance, it is critical to conduct mode of action studies in parallel to ensure that the pipeline consists of compounds inhibiting a diverse set of chemically and genetically validated targets. One such cautionary tale is provided by studies to repurpose clofazimine, a leprosy therapeutic identified as a *Cryptosporidium* hit from a phenotypic screen (Love et al., 2017). How clofazimine works to kill *Cryptosporidium* is not understood. While effective *in vitro* and in mouse models of cryptosporidiosis, clofazimine lacked efficacy in a recent clinical trial of HIV patients (Iroh Tam et al., 2021). Mode of action tools may help us understand why this treatment failed in a clinical setting and may inform how we can avoid future clinical failures for other promising anti-cryptosporidial compounds in development.

Using the target-compound pair *Cp*KRS and DDD01510706, we developed new mode of action tools for *Cryptosporidium*. Aminoacyl-tRNA synthetases have been targets of interest for treatment of parasitic diseases for over 20 years (Pham et al., 2014) and are the focus of several on-going target-based drug discovery programs for cryptosporidiosis (Palencia et al., 2016; Jain et al., 2017; Vinayak et al., 2020; Hasan et al., 2021). First, we established TPP to support unbiased target identification in *Cryptosporidium*. *Cp*KRS was determined as the sole target of DDD01510706 by both ΔT_m_ and NPARC analysis. In the future, TPP can be applied to several promising therapies in development for cryptosporidiosis that lack a clear molecular target. This includes compounds such as MMV665917, which is effective in both *C. parvum* and *C. hominis in vivo* animal models (Stebbins et al., 2018; Lee et al., 2019).

One caveat of our current TPP approach is that we are limited to using sporozoite parasites to generate the protein lysate. Sporozoites are used because they can be obtained in high numbers without host material. Compounds whose molecular target is specifically expressed in other life cycle stages, such as sexual stages (Jumani et al., 2019), would not be amenable for study by the method we report here. Unlike other apicomplexan parasites, currently we are not able to physically separate intracellular *Cryptosporidium* from their host cell. This means lysate made from intracellular *Cryptosporidium* life cycle stages will contain a large amount of host cell derived protein. For compounds that target parasite proteins, it may be difficult to generate a high coverage of the predicted parasite proteome for target identification by TPP. However, for compounds predicted to act via a host mechanism, establishing TPP for infected host cells may prove very powerful.

These limitations highlight the need to invest in the development of further mode of action tools, as no single tool will successfully identify every target. This could be expanded to include pull-down with chemical probes to identify molecular targets from parasite lysate. In our experience to date, this also has considerable challenges similar to TPP including obtaining enough high-quality lysate, sufficient information around structure-activity-relationship to design linker compounds avoid interfering with the compound-target interaction, and the requirement for chemistry expertise to generate linker compounds. More work with well validated tool compounds is required to establish protocols before this approach can be applied for target identification in *Cryptosporidium*. Advancements in *Cryptosporidium in vitro* culture may allow for evolution of resistance similar to *Plasmodium*. Many groups are pursuing continuous *in vitro* culture systems through use of intestinal organoid tissues, creating complex 3D cultures, or applying a more realistic gut environment through modifying nutrients, oxygen levels, etc.

We used genetic approaches to validate lysyl-tRNA synthetase (KRS) as an essential drug target. We were unable to knockout KRS, and a conditional strain was unable to infect mice when KRS was knocked down. Previous work established conservation of drug binding between *Pf*KRS and *Cp*KRS and identified specific residues in the *Pf*KRS binding pocket that when mutated confer resistance to DDD01510706 (Milne et al., 2022). When we engineered similar mutations in *Cp*KRS (KRS^R^) we observed resistance to DDD01510706. Mutations at the active site of other aminoacyl tRNA synthetase targets have been similarly employed to demonstrate on-target activity (Vinayak et al., 2020; Hasan et al., 2021). The disadvantage of mutating key amino acid residues is that it requires extensive knowledge of the drug-target interaction. Target overexpression requires comparatively less information about the target. Expression of an additional copy of *Cp*KRS driven by a strong, constitutive promoter also conferred resistance to DDD01510706. By generating resistance-conferring modifications with different genetic approaches, we provide strong evidence of on-target activity. These genetic and chemical tools are critical resources for mode of action studies that can be readily applied to other targets and anti-cryptosporidial compounds in development.

Although DDD01510706 is not being progressed due to toxicity issues, it is a very useful tool compound. We replicated *in vivo* treatment with DDD01510706 in the acute efficacy model of cryptosporidiosis and found that treatment lowered infection to the level of detection. Because of lack of a simple, continuous *in vitro* culture for *Cryptosporidium*, we are dependent on *in vivo* models to generate and propagate transgenic *Cryptosporidium* strains. This dependence on *in vivo* models necessitates that selection agents must be effective *in vivo*, well tolerated in mice, and affordable to purchase or synthesize in quantities required for *in vivo* work. Currently, there is only a single drug and corresponding selection marker that meets these criteria: namely paromomycin and Neo^R^. Through our mode of action work reported here, we determined that DDD01510706 and KRS containing an A309L mutation, KRS^R^, also fulfil these criteria.

KRS^R^ is a second drug marker for genetic modification of *Cryptosporidium*. KRS^R^ is interchangeable with Neo^R^ as a selection marker. Either can be inserted at a locus of interest under a constitutive promoter, independent of the endogenous wild type *KRS* gene. The selection agent is provided *in vivo* starting day one post infection, for the duration required to kill unmodified wild type parasites. The discovery of a second selection marker greatly expands the possibilities for *Cryptosporidium* reverse genetics like complementation that require two selection markers (Gubbels et al., 2008). It also enables sophisticated genetic modification strategies often used in other apicomplexan parasites, including conditional gene expression systems such as split-Cas9 (Li et al., 2022) and diCre (Andenmatten et al., 2013). This second selection marker will inspire the next generation of genetic tool development for study of this important diarrheal pathogen.

In addition to utility as a selection marker, KRS^R^ can be used in combination with Neo^R^ to select for experimental genetic crosses. Completion of the life cycle occurs *in vivo* in immunocompromised mouse models and natural hosts, and has been reported *in vitro* in complex culture models such as organoids (Heo et al., 2018; Wilke et al., 2019). Genetic crossing observed in these models follows Medelian inheritance, producing only a small proportion of hybrid parasites. Here, we describe a highly efficient *Cryptosporidium* experimental genetic cross using two selection markers. Mice infected with both mScarlet-KRS^R^ and mNeon-Neo^R^ resistant strains, and that were treated with both selection agents, shed >99% hybrid progeny oocysts. When excysted, we observed that individual sporozoites are hybrids, and express both mScarlet and mNeon.

KRS^R^ enables forward genetic approaches in *Cryptosporidium*. Forward genetics has been powerful in *Toxoplasma* for deciphering mechanisms of virulence (Grigg et al., 2001; Su et al., 2002; Taylor et al., 2006), drug resistance (Behnke et al., 2015), and cell cycle progression (Gubbels et al., 2008). Several unexplored areas of *Cryptosporidium* biology are highly suited to investigation using forward genetics including virulence, host-specificity observed by different species, mechanisms of sexual recombination, and evolution of drug resistance. From a single *Cryptosporidium* mouse infection, we can produce hundreds of thousands of hybrid oocysts for analysis, several orders of magnitude larger than progeny analysed in previous *Toxoplasma* crosses. Some challenges remain, like cloning *Cryptosporidium*, however there are several advantages to performing genetic crosses in *Cryptosporidium*. The study of sexual stages and genetic crossing in other apicomplexans requires *in vivo* models that are complex and hard to access (felines for *Toxoplasma* and mosquitos and humanized mice for *Plasmodium falciparum*). However, no additional *in vivo* models are required for *Cryptosporidium*. Additionally, the small size of the *Cryptosporidium* genome (9.1 Mb) makes analysis of hybrids by whole genome sequencing feasible. *Cryptosporidium* represents an accessible model to study apicomplexan genetic recombination, and how selection pressures (including drug treatment) may drive recombination and population diversity.

Despite its global health impact, there are no effective treatments for cryptosporidiosis and a staggering lack of validated drug targets. Mode of action studies and target identification are critical for the advancement of drug discovery efforts. These studies also produce tool compounds, new techniques, and technology that add to our understanding of the basic biology of parasites. Through our mode of action studies on lysyl-tRNA synthetase we generated KRS^R^, which greatly expands our capacity to investigate *Cryptosporidium* basic biology by immediately expanding the tools for reverse genetics. KRS^R^ also opens the door for forward genetics, an approach that promises to transform our understanding of *Cryptosporidium* virulence, host-specificity, and mechanisms of sexual recombination.

## Data availability statement

The datasets presented in this study have been deposited to the ProteomeXchange Consortium via the PRIDE partner respository with the dataset identifier PXD037942 at https://www.ebi.ac.uk/pride/archive/.

## Ethics statement

The animal work was reviewed and approved by the University of Dundee Welfare and Ethical Use of Animals Committee and operates under a Project Licence awarded by the UK Home Office.

## Author contributions

JCH: Conceptualization, methodology, investigation, data curation, visualization, writing - original draft. VC-L: Methodology, investigation, software, formal analysis. SS: Conceptualization, methodology, investigation, visualization. BC and LR: Methodology, investigation. RB: Investigation. GMJH and EMS: Validation, investigation. BB: Resources. SW: Funding acquisition, supervision, conceptualization, resources, writing- review and editing. MCP: Funding acquisition, supervision, conceptualization, resources, visualization, project administration, writing- original draft. All authors contributed to the article and approved the submitted version
