# Supplementary material for: Mode of action studies confirm on-target engagement of lysyl-tRNA synthetase inhibitor and lead to new selection marker for Cryptosporidium
Source: Front Cell Infect Microbiol. 2023 Aug 4;13:1236814. doi: 10.3389/fcimb.2023.1236814 (PMC10436570; doi:10.3389/fcimb.2023.1236814)
Supplement: Supplementary file 1 [file DataSheet_1.docx]

Supplementary Material

Mode of action studies confirm on-target engagement of lysyl-tRNA synthetase inhibitor and lead to new selection marker for *Cryptosporidium*

**Jack C Hanna^1,3^, Victor Corpas-Lopez^1%^, Simona Seizova^1%^, Beatrice L Colon^1^, Ross Bacchetti^1, 4^, Grant MJ Hall^1^, Emma M Sands^1^, Lee Robinson^1^, Beatriz Baragaña^1,2^, Susan Wyllie^1^*, Mattie C Pawlowic^1^***

**^1^Wellcome Centre for Anti-Infectives Research, School of Life Sciences, University of Dundee, Dundee, DD1 5EH, United Kingdom**

**^2^Drug Discovery Unit, School of Life Sciences, University of Dundee, Dundee, DD1 5EH, United Kingdom**

**^3^Current address: Wellcome Centre for Integrative Parasitology, School of Infection and Immunity, University of Glasgow, Glasgow, G12 8TA, United Kingdom**

**^4^Current address: Scottish Microbiology Reference Laboratories, Glasgow Royal Infirmary, Glasgow, G31 2ER, United Kingdom**

**^%^Equal contribution: these authors contributed equally to this work**

*** Correspondence:
Mattie C Pawlowic; Susan Wyllie**[**mcpawlowic@dundee.ac.uk**](mailto:mcpawlowic@dundee.ac.uk)**,** [**s.wyllie@dundee.ac.uk**](mailto:s.wyllie@dundee.ac.uk)

**Supplementary Table 1. Protein targets identified by TPP (as listed in Figure 1D).**

| **Data analysis method** | **CryptoDB gene ID** | **Protein description** |
| --- | --- | --- |
| ΔT_m_ and NPARC | cgd4_2370 | Lysyl-tRNA synthetase (KRS) |
| ΔT_m_ | cgd4_420 | Gdt1 family |
|  | cgd5_1530 | Uncharacterized protein |
| NPARC | cgd8_2550 | Uncharacterized transmembrane protein |
|  | cgd6_5520 | Metalloenzyme, LuxS/M16 peptidase-like |
|  | cgd4_3100 | Adenylyl cyclase class-3/4/guanylyl cyclase |
|  | cgd8_2780 | Integral membrane protein EMC3/TMCO1-like |
|  | cgd5_3243 | Uncharacterized protein |
|  | cgd1_2950 | XAP5 protein |
|  | cgd3_250 | Serine/threonine-protein phosphatase |

**Supplementary Table 2. Oligonucleotides used in this study.**

| **Name** | **DNA Sequence (5´ – 3´)** |
| --- | --- |
| CpKRS qPCR F | GAGCACGACATTAGCACTGG |
| CpKRS qPCR R | TCAGACCAGAAATCGCCGTA |
| Cp18S_F | ATGACGGGTAACGGGGAAT |
| Cp18S_R | CCAATTACAAAACCAAAAAGTCC |
| KRS guide F | GTTGGTCTCTAGTTGCTTCTATTAG |
| KRS guide R | AAACCTAATAGAAGCAACTAGAGAC |
| KRS-KO homology F | GGACGCAGGCAGACCGTTTTATCCACACAAGTTTAAAATTTCGATGTCGCTGGGGAAACTAAATATACTGAAATTCGG |
| KRS-KO homology R | GGAATGTTTAAAAATTCTCTAGTTGCTTCTATTAGTGATTGAAACTTTCAATTAAGATAAAAAGAAAAACTTAATCGATACTATCCTACACGCC |
| KRS-Neon homology F | CTATGAGAAACGTTAAACAAAATGCCCAACATAGCAACCAACATAGCGGAAATGTGTCCAAGGGCGAGGAGGAC |
| KRS-Neon homology R | GGAATGTTTAAAAATTCTCTAGTTGCTTCTATTAGTGATTGAAACTTTCAATTAAGATAAAAAGAAAAACTTAATCGATACTATCCTACACGCC |
| KRS-Neon 5´ integration F | GAGACATTCTGTCTTGCATTAGAGCACGG |
| KRS-Neon 5´ integration R | CGAGTTGGTCATCACCGGG |
| KRS-Neon 3´ integration F | GCCGGTCTTGTCGATCAAGATGATCTTG |
| KRS-Neon 3´ integration R | GCTAAATGGCTCTGATTTAGGATTTC |
| TK-ORF F | ATGGCAAAATTATACTTTTACTATTCAGCAATGAATGC |
| TK-ORF R | TTAGAAATTGTATTCTTCACAATTAATTATATGATGTTTTCTGC |
| KRS-DDD homology F | GAGAAACGTTAAACAAAATGCCCAACATAGCAACCAACATAGCGGAAATCCTAGGTACCCGTACGACGTCCCGG |
| KRS-DDD homology R | GGAATGTTTAAAAATTCTCTAGTTGCTTCTATTAGTGATTGAAACTTTCAATTAAGATAAAAAGAAAAACTTAATCGATACTATCCTACACGCC |
| KRS-DDD 5´ integration F | GACTCTCAGGAGAATATCGATTTTATG |
| KRS-DDD 5´ integration R | GGAACATCGTAAGGATACGCATAATCGGGCACATCATAGG |
| KRS-DDD 3´ integration F | GCCGGTCTTGTCGATCAAGATGATCTTG |
| KRS-DDD 3´ integration R | GCTAAATGGCTCTGATTTAGGATTTC |
| OE-KRS backbone HiFi F | GGCTCCGGAGGCCAGAGGGCGACGAACTTCAGCCTGCTCAAGCAGGCGGGTGATGTGGAGGAGAACCCAGGTCCGATGGTCTTCACACTCGAAGATTTCGTTGGGGAC |
| OE-KRS backbone HiFi R | CAATATGTAGCTTAAACAAGGCATGGCTAAACTTCAATACAAAAATCTCAAATAAATTTG |
| OE-KRS insert HiFi F | GCCATGCCTTGTTTAAGCTACATATTGGCGGCCCTCGTTGAGACGATAATCAGAC |
| OE-KRS insert HiFi R | GTCGCCCTCTGGCCTCCGGAGCCTCCGCCACCTGATCCGCCACCTCCGCTCCCACCACCATTTCCGCTATGTTGGTTGCTATGTTGGGCATTTTG |
| OE-KRS homology F | CTTTAAAGCAATAATATCACTCATACCTACTGCAAATAAGATTGGAAATACTGGGGAAACTAAATATACTGAAATTCGGTAG |
| OE-KRS homology R | GACTCCTTTTTAGGCACTTTCAAGAGGCGCCATAGCTGCGCCAAATTTTGCAATTAAGATAAAAAGAAAAACTTAATCGATACTATCCTACACGCCACG |
| OE-KRS 5´ integration F | CAATTTATCTGGTGCATCTTGATTTTG |
| OE-KRS 5´ integration R | GAGTTTGGTGTGCAGGCGCCTGATCG |
| OE-KRS 3´ integration F | GATCTTCTTTCTTCTCACCTTGCTCC |
| OE-KRS 3´ integration R | CAAAGTATCTTGATCTTTTGCTTCAC |
| Tubulin ORF F | CTAGTTATCCTTGTTCATTGAATTCTC |
| Tubulin ORF R | TGAGCTCAAAAATATAAGATGGCAC |
| A309L KRS HiFi F | GGAAGTGGAGGACGGGAATTCTTTAGGAATGAAGGAATAGATTTAACACACAACCC |
| A309L KRS HiFi R | ACGTCGTACGGGTACCTAGGATTTCCGCTATGTTGGTTGCTATGTTGGGC |
| A309L-KRS homology F | TATGAAATAGGAAAAAACTTTAGGAATGAAGGAATAGATTTAACACACAATCCTGAGTTCACATTAATGGAGTTTTACATGGCATATGCTGATTATTATG |
| A309L-KRS homology R | GTAACTAACTCTTCGCTAGTGTTAGTTGTTGAGAAACCACCGCAATCTAACTCAATTAAGATAAAAAGAAAAACTTAATCGATACTATCCTACACGCCACG |
| A309L guide F | GTTGACTCCATTGCTGTGAACTCA |
| A309L guide R | AAACTGAGTTCACAGCAATGGAGT |
| A309L-KRS 5´ integration F | TACGGCGATTTCTGGTCTGA |
| A309L-KRS 5´ integration R | GGATACGCATAATCGGGCAC |
| A309L-KRS 3´ integration F | GCCGGTCTTGTCGATCAAGATGATCTTG |
| A309L-KRS 3´ integration R | GCTAAATGGCTCTGATTTAGGATTTC |
| FNN backbone HiFi F | GGCCAGAGGGCGACGAACTTCAGCCTGCTCAAGCAGGCGGGTGATGTGGAGGAGAACCCAGGTCCGATGGTCTTCACACTCGAAGATTTCG |
| FNN backbone HiFi R | GATGTTCTTGGCGTCCTCCATGTCGACGGCTAAACTTCAATAC |
| FNN insert HiFi F | ATGGAGGACGCCAAGAACATCAAGAAGGGACCAGCCCCCTTC |
| FNN insert HiFi R | GGCTGAAGTTCGTCGCCCTCTGGCCTCCGGAGCCTCCGCCACCTGATCCGCCACCTCCGCTCCCACCACCCTTATACAATTCGTCCATCCCCATCAC |
| FNN homology F | TTTAAAGCAATAATATCACTCATACCTACTGCAAATAAGATTGGAAATACTGGGGAAACTAAATATACTG |
| FNN homology R | ACTCCTTTTTAGGCACTTTCAAGAGGCGCCATAGCTGCGCCAAATTTTGCGGAACTTCACCGGTAATTAAGATAAAAAG |
| FNN 5´ integration F | CAATTTATCTGGTGCATCTTGATTTTG |
| FNN 5´ integration R | GATGTTCTTGGCGTCCTCCATGTCGACGGCTAAACTTCAATAC |
| FNN 3´ integration F | GTGGTCGTCTTGGTGTCGCCGACC |
| FNN 3´ integration R | GCATCCTCCTTTGTAACCTTTATTC |
| KRS^R^ or Neo^R^ Homology F | GCGCCAAATTCCTGTTATATGATAAATATTAATTAGATATCGACTATTTCTGGGGAAACTAAATATACTG |
| KRS^R^ or Neo^R^ Homology R | GCTCATATGATTTAAATTTCAAAATGAAAAAACCAAAATAAATTTACAATTTAATATCACGGAAGGGG |
| IMPDH-KRS^R^ 5´ integration F | GCTTAGTGGATTGGCGCCAACATATTATTTTATTCAAGTG |
| IMPDH-KRS^R^ 5´ integration R | GAGTTTGGTGTGCAGGCGCCTGATCG |
| IMPDH-KRS^R^ 3´ integration F | GACTCTCAGGAGAATATCGATTTTATG |
| IMPDH-KRS^R^ 3´ integration R | CAAACTGGTGACTATATTTGTTTAACAAAC |
| IMPDH-Neo^R^ 5´ integration F | GCTTAGTGGATTGGCGCCAACATATTATTTTATTCAAGTG |
| IMPDH-Neo^R^ 5´ integration R | GAGTTTGGTGTGCAGGCGCCTGATCG |
| IMPDH-Neo^R^ 3´ integration F | GCCGGTAGAGACTGGCTTCTTTTAGG |
| IMPDH-KRS^R^ 3´ integration R | CAAACTGGTGACTATATTTGTTTAACAAAC |
| IMPDH ORF F | GATGTAATTGTTGGGAATGTTGTAACAGAAGAAGCAAC |
| IMPDH ORF R | GCAGGATCTTAGTCCTCCAACAAGCTGATATACTACACCTTCCATTTCACC |

*Pf*KRS1 IC_50_ 0.015 µM

*Cp*KRS1 IC_50_ 0.13 µM

Nluc *Cp* EC_50_ 2.5 µM

HsKRS IC_50_ 1.8 µM

HepG2 EC_50_ 49 µM

MLM CLi 1ml/min/g

CHI-logD 1.7 (clogP 2.4)

**Supplemental Figure 1. Structure of Late Lead Compound 5, DDD01510706.**

DDD01510706 has activity against *Cryptosporidium* both *in vitro* and *in vivo*. Data from Baragana B*, et al.* Lysyl-tRNA synthetase as a drug target in malaria and cryptosporidiosis. *Proc Natl Acad Sci U S A* **116**, 7015-7020 (2019). Compound available to request from: https://mrcppureagents.dundee.ac.uk/

**Supplemental Figure 2. Design of CpKRS-tagged transgenic strains.**

**A)** Schematic of the strategy to knock out *CpKRS* open reading frame (ORF). To avoid interruption of the upstream gene (cgd4_2373, black arrow), homology (grey region) was chosen to result in the deletion of 86% of the ORF (location of gRNA, black arrowhead). Scale bar indicates 1000 bp. **B)** KRS-mNG strain was generated by fusing mNeonGreen to *Cp*KRS at the C-terminus with a Cp*Aldolase* 3´ untranslated region (UTR), followed by the NanoLuciferase-Neomycin resistance fusion (Nluc-Neo^R^) under the control of the *CpEnolase* untranslated regions (UTRs) (EnoP and Eno 3´ UTR; 3xHA, purple). Attempts at epitope tagging *Cp*KRS with a 3x hemagglutinin (HA) tag were successful, but did not produce a bright enough signal to enable analysis (data not shown), so this fluorescent fusion was generated instead. **C)** DNA was extracted from wild type (WT) and KRS-mNG strains. PCR amplification of the 5´ integration (5´ Int) and 3´ integration (3´ Int) sites confirm correct insertion of repair template as depicted in **B**. PCR amplification of an independent loci (thymidine kinase, *CpTK*) serves as PCR positive control. **D)** A conditional knockdown strain was generated by fusing *Cp*KRS at the C-terminus to a dihydrofolate reductase degradation domain (DDD, magenta), followed by the Nluc-Neo^R^ resistance fusion under the control of the *CpEnolase* UTRs. **E)** Amplification of the 5´ and 3´ integration sites of the KRS-DDD strain confirm correct insertion of repair template as depicted in **D**.

**
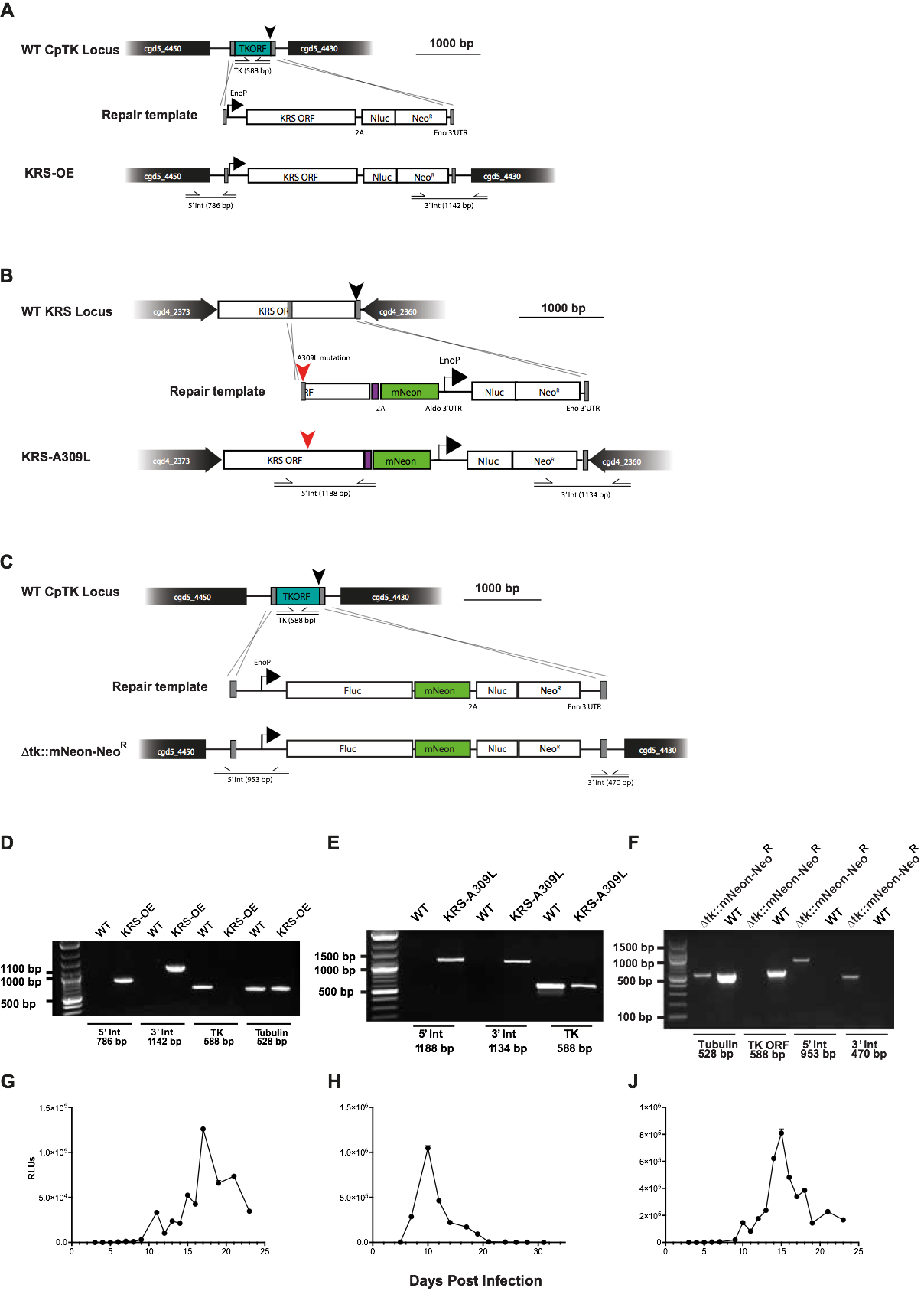
**

**Supplemental Figure 3. Generation of additional strains utilized in drug assays.**

**A)** Generation of KRS-OE strain that overexpresses *CpKRS* by insertion of an additional copy under the constitutive *CpEnolase* promoter at the *thymidine kinase* locus (*CpTK*, teal). **B)** Generation of a *Cryptosporidium* strain KRS-A309L with an amino acid substitution in the endogenous *Cp*KRS to generate resistance (A309L, red arrow). KRS-A309L also drives expression of mNeon. **C)** To generate the Δ*tk*::mNeon-Neo^R^ reporter strain, a cassette containing firefly luciferase (Fluc), mNeon, and an Nluc-Neo^R^ resistance fusion under the control of the *CpEnolase* promoter was inserted at the *CpTK* locus. **D)** PCR confirms the correct 5´ and 3´ integration of the repair construct depicted in **A** and deletion of *CpTK*. *CpTubulin* serves as PCR positive control. **E)** PCR confirms the correct 5´ and 3´ integration of the repair construct in **B**. **F)** PCR confirms correct integration of the repair cassette at the *CpTK* locus as illustrated in **C**. **G)** NanoLuciferase expression from feces collected from IFN-γ KO mice infected with OE-KRS, **H)** KRS-A309L, and **J)** Δ*tk*::mNeon-Neo^R^ reporter strains. RLUs, relative luminescence units; RLUs/2 mg of fecal material plotted.

**
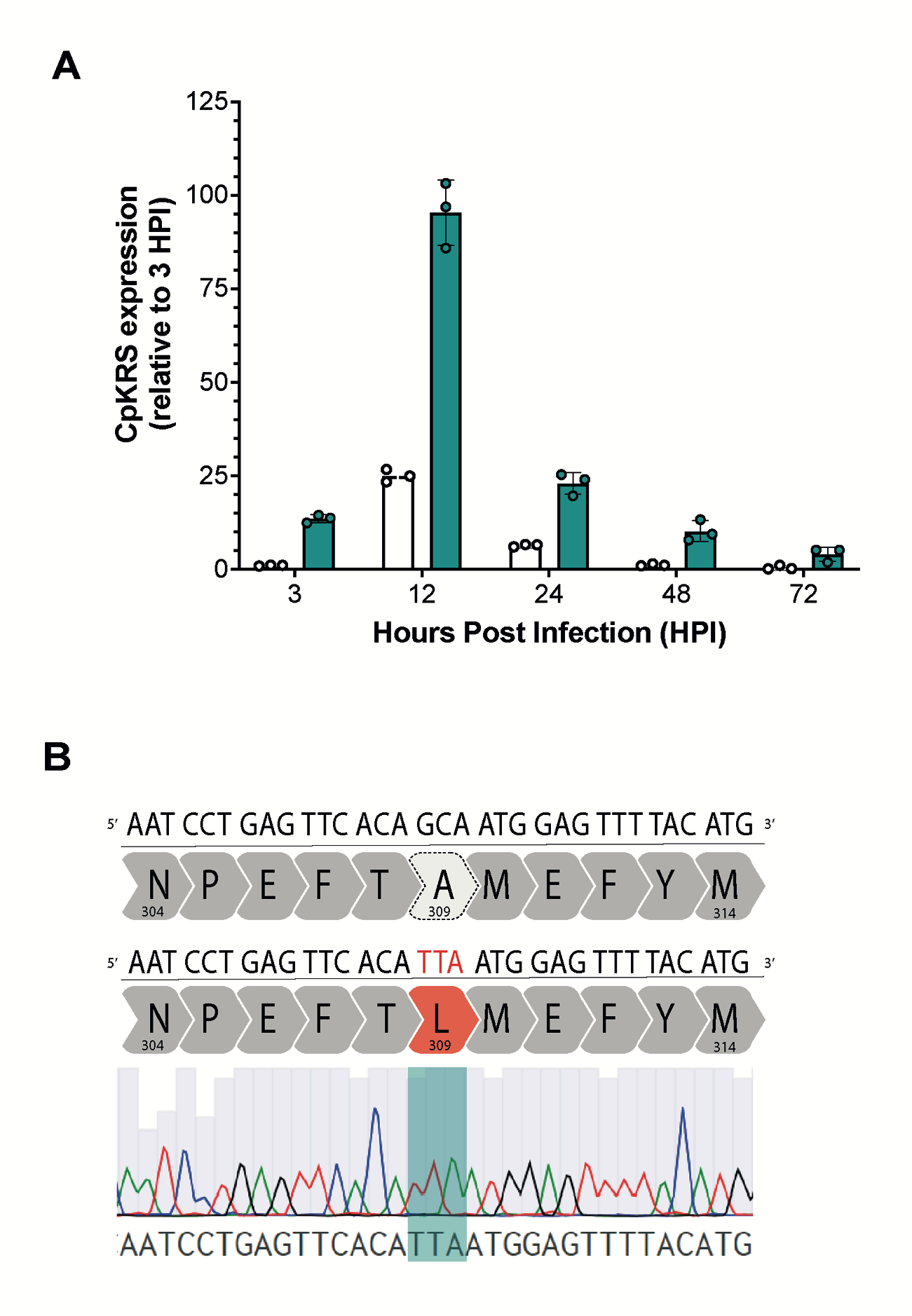
**

**Supplemental Figure 4. Validation of CpKRS-modified strains.**

**A)** Wild type (WT) and KRS-OE parasites were cultured in HCT-8 cells for up to 72 hours. At the indicated time points, total RNA was extracted, cDNA was prepared, and RT-qPCR performed on *CpKRS* to quantify and compare transcript abundance between strains. *CpKRS* expression relative to WT expression at 3 HPI was determined using ΔΔCt method. Mean ± SD. One biological replicate and three technical replicates. **B)** Total DNA was extracted from A309L-KRS parasites and the modified region of *CpKRS* was PCR amplified, cloned into a TOPO-TA vector (ThermoFisher Scientific), and transformed into bacteria. Ten clones were Sanger sequenced and all were found to harbor the correct base substitutions that result in the A309L amino acid substitution, as designed.

**Supplemental Figure 5. Selectivity window of DDD01510706.**

HCT-8s are a human intestinal epithelial cell line used for *Cryptosporidium in vitro* culture. HCT-8 cell survival (teal) declines at higher doses of DDD01510706, indicating host cell toxicity. Parasite death (magenta) occurs at doses where HCT-8 survival is not impacted by DDD01510706. *Cryptosporidium* EC_50_ (7.5 μM) and EC_90_ (28 μM) are indicated by the dotted lines. Mean ± SEM; 3 biological replicates, each with a minimum of 3 technical replicates.

**Supplemental Figure 6. Treatment with DDD01510706 reduces *Cryptosporidium* infection**.

IFN-γ KO mice were infected with a reporter parasite strain that express NanoLuciferase (**Supplemental Figure 3C**; also used for all *in vitro* drug assays). Mice were age and sex matched and grouped 6 mice per cage. Mice were treated by gavage once daily with vehicle (black circle) or with DDD01510706 (20 mg/kg; white circle). Treatment lasted for 7 days, from day 6 to day 12 post infection (gray box). Fecal samples were collected daily from the entire cage and infection level was determined by NanoLuciferase assay (RLUs, relative luminescence units; RLUs/2 mg of fecal material plotted). Mean ±SD ; 3 biological replicates, each with 3 technical replicates. Limit of quantification is 10^3^ RLU; dotted line.

**
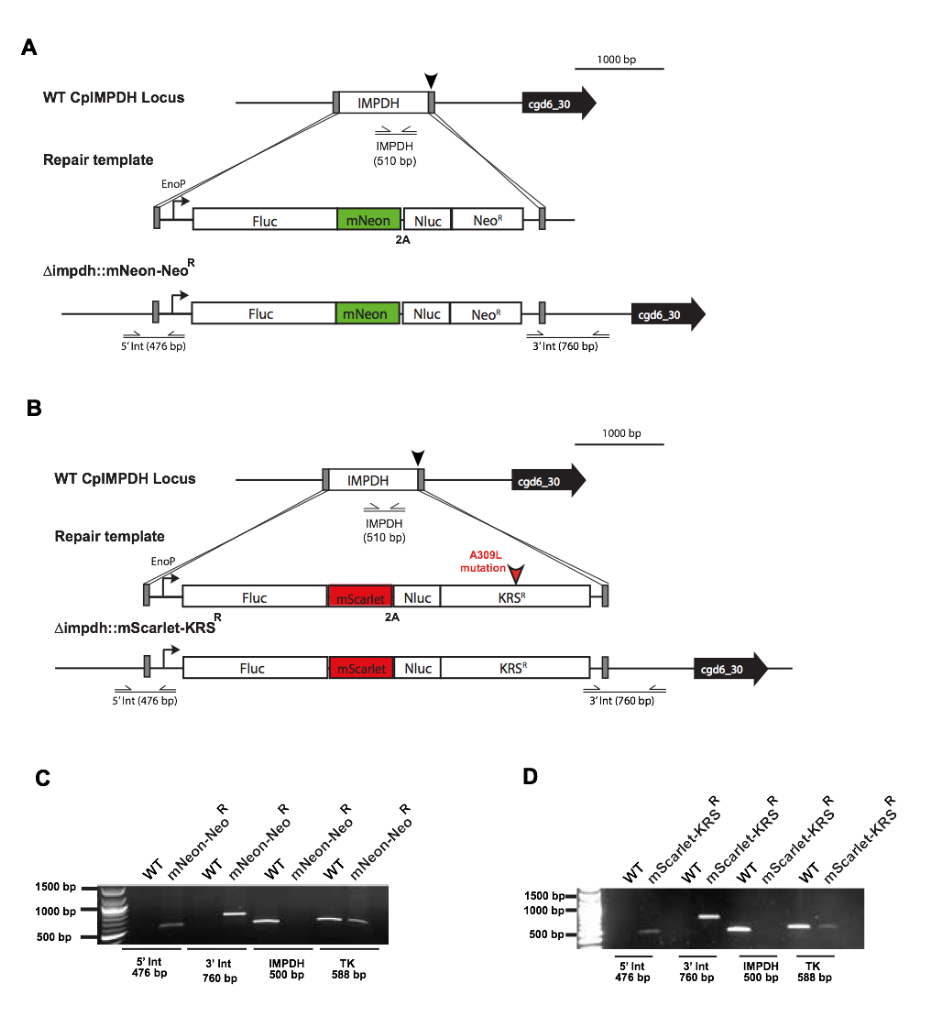
**

**Supplemental Figure 7. Design for generation of KRS^R^ drug marker transgenic strain.**

**A)** The *CpIMDPH* locus was targeted for replacement by CRISPR/Cas9 with the repair cassette illustrated in **Supplemental Figure 3C** to generate the Δ*impdh*::mNeon-Neo^R^ strain. This strain expresses Fluc, mNeon, and Nluc-Neo^R^ and is resistant to treatment with paromomycin. This strain can be selected for *in vivo* by treating mice with 16 g/L in the water bottle. See **Supplemental Figure 8A and 8E** for infection curves. **B)** The *CpIMPDH* locus was targeted for replacement by CRISPR/Cas9. The repair cassette used in **A** was modified such that mNeon was replaced with mScarlet and Neo^R^ was replaced with KRS-A309L (KRS^R^) to generate the Δ*impdh*::mScarlet-KRS^R^ strain. This strain expresses Fluc, mScarlet, Nluc-KRS^R^ and is resistant to treatment with DDD01510706. This strain can be selected for *in vivo* by treating mice with 20mg/kg for 7 days by oral gavage. See **Supplemental Figure 8B-D and F-H** for infection curves. **C)** PCR confirmation of the correct 5´ and 3´ integration of the construct shown in **A** as well as the deletion of the *CpIMPDH* gene. Amplification of *CpTubulin* was included as a positive control. **D)** PCR confirmation of the correct 5´ and 3´ integration of the construct shown in **B** as well as the deletion of the *CpIMPDH* gene. Amplification of *CpTubulin* was included as a positive control.

**
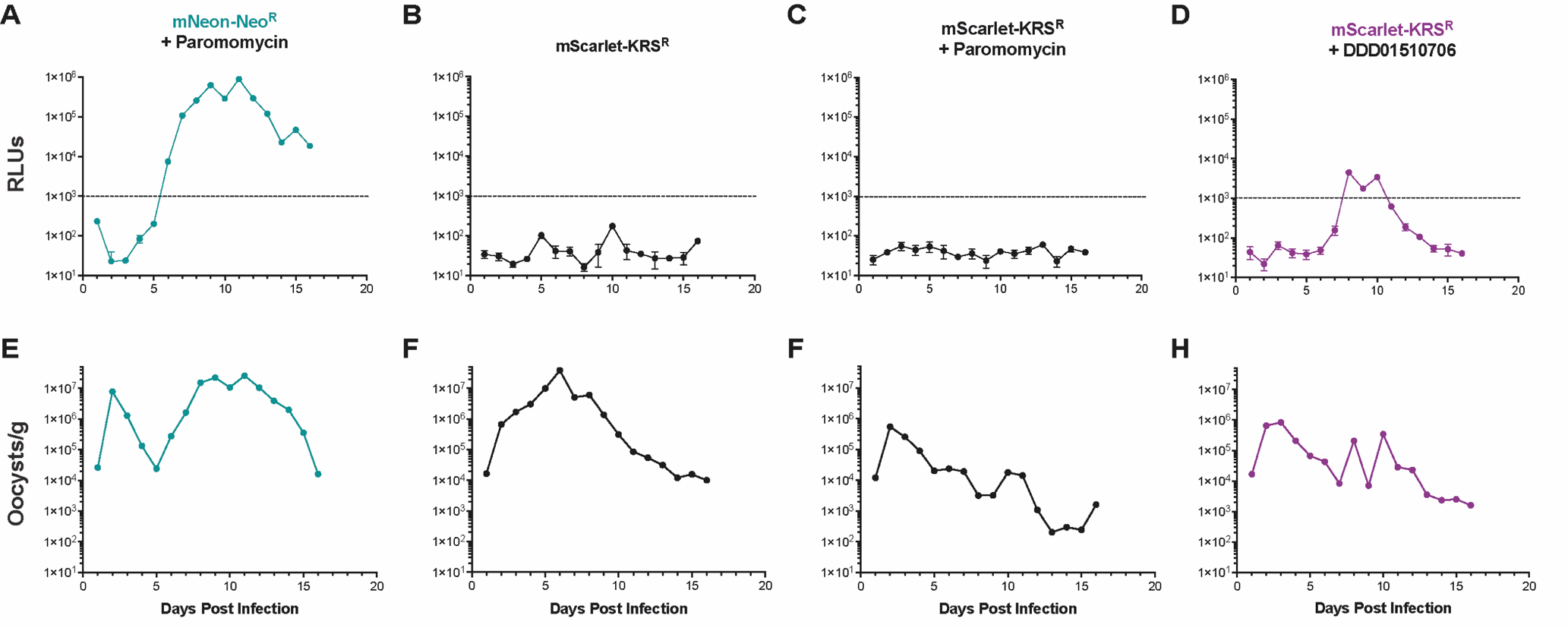
**

**Supplemental Figure 8. Successful selection of new *Cryptosporidium* transgenic strain (transfection with KRS^R^ drug marker and *in vivo* treatment with DDD01510706).**

Wild type *Cryptosporidium* sporozoites were transfected with CRISPR/Cas9 constructs designed to target the **A)** *CpIMPDH* locus for replacement with mNeon-Neo^R^ (see **Supplemental Fig 7A**) or **B-D)** mScarlet-KRS^R^ (see **Supplemental Figure 7B**). As illustrated in **Figure 4A**, IFN-γ KO mice (4 per cage, age and sex matched) were infected by gavage. Starting day 1 post infection, transgenic parasites were selected either by treatment with paromomycin delivered in the water bottle (“+ Paromomycin”) at 16 g/L, or by gavage with DDD01510706, dosed daily at 20 mg/kg for a total of 7 days (“+ DDD01510706”). Fecal samples were collected from each cage and analyzed for infection determined by Nluc (**A-D**) or qPCR (**E-H**). Expression of NanoLuciferase (RLUs, relative luminescence units; RLUs/2 mg of fecal material plotted; average and SD of three technical replicates). RLU > 1000 indicate successful infection of transgenic parasites, dotted line.

**
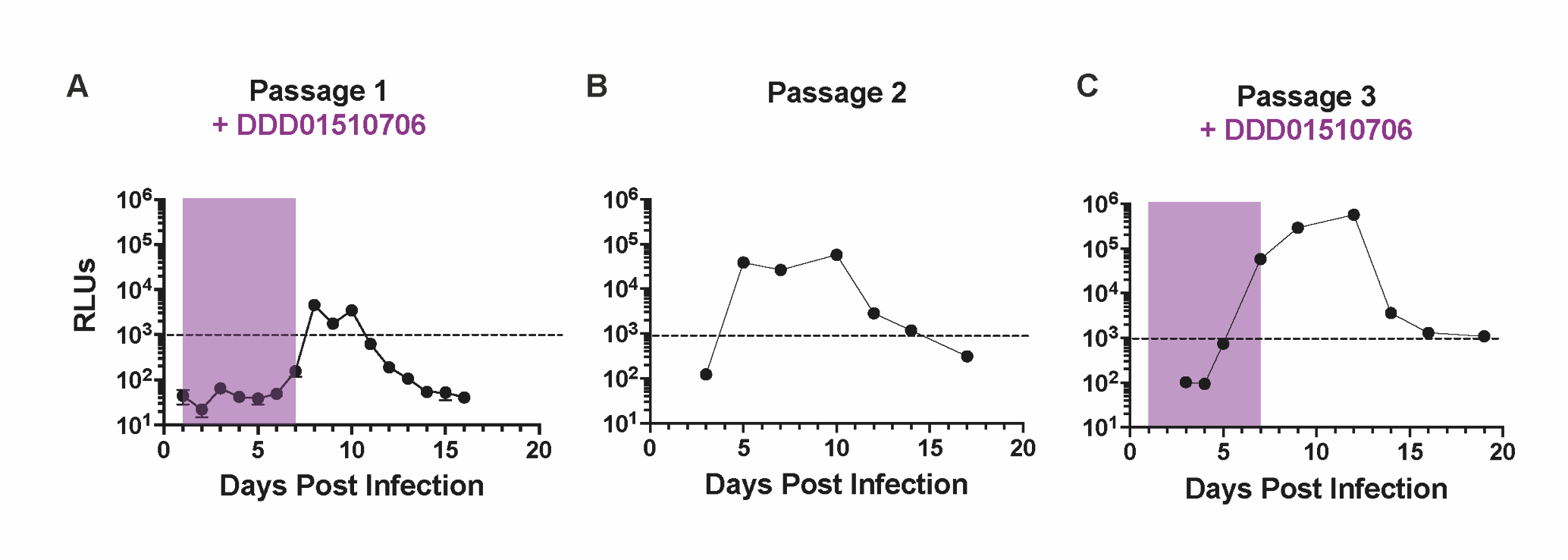
**

**Supplemental Figure 9. Passaging and selection strategy for mScarlet-KRS^R^ strain.**

**A)** Infection curve of mScarlet-KRS^R^ determined by NanoLuciferase assay (**Supplemental Figure 8D)**. IFN-γ KO mice (4 per cage) were infected with transfected sporozoites and mice were treated with 20 mg/kg DDD01510706 starting day 1 post infection, for a total of 7 days (purple box). **B)** A slurry was prepared from feces collected from days with RLU > 1000 from the first passage and used to infect a cage of mice in a second passage. **C)** Oocysts were purified from fecal samples collected in the second passage were used to infect a third cage. Mice were treated with 20 mg/kg DDD01510706 starting day 1 post infection, for a total of 7 days (purple box). Oocysts from the third passage were utilized in the genetic cross experiment illustrated in **Figure 4**.

**Supplemental Figure 10. FACS gating strategy to quantify fluorescent oocysts.**

**A)** Initial gate used to select *Cryptosporidium* oocysts for analysis. **B)** Second gate to capture single oocysts. **C)** Final gate to analyze fluorescent oocysts. **D)** Samples from day 10 post infection from crossing experiment (**Figure 4C**) illustrate quantification of oocysts positive for mNeon, mScarlet, or both (purple). Strains used for crossing experiment (**Figure 4B**) served as controls for gating in **C**: mNeon-Neo^R^ (**E**) and mScarlet-KRS^R^ (**F**). Percentage of total events indicated in corresponding gate.

**Supplemental Figure 11. Hybrid oocysts are made up of hybrid sporozoites.**

Oocysts resulting from crossing experiment (see **Figure 4G**) were excysted and live-imaged. All sporozoites that emerge from hybrid oocysts express both mNeon (**A**) and mScarlet (**B**). Scale bar 5 μm (**C**).
